# Dr. Reid's Case of Trismus

**Published:** 1803-10-01

**Authors:** J. Reid

**Affiliations:** Southampton-Row


To Mr. WARD, Surgeon, Manchester.

SIR,

AS you have expressed to me a wish, with which others perhaps may sympathize, to be acquainted more particularly with an instance of Trismus, alluded to in one of my periodical Reports of diseases in the Monthly Magazine, I have thought it right to communicate to the Editors of the Medical and Physical Journal; and, in case of their permission, to the public, a brief abstract of the case, as it was taken down during my attendance upon the patient.

I am, &c.

*Southampton-Row,* J. REID.

*July* 16, 1803.

---

TRISMUS TRAUMATICA.

Ap. 27, 1803. Landeman Jues, æt. 24, a marine, has left the service nearly a twelve month, having received a severe wound in his leg, which, within the last fortnight, has given him very great pain and uneasiness. A few days ago he was attacked with the usual symptoms of a *cold,* and slight fever, which confined him to bed; a day or two after which he was seized with a violent pain and stiffness of the jaw, especially severe on the left side, which has since greatly increased, and at present, (the seventh day after the attack of the disease) his jaw is *locked,* but not so completely as to prevent broths and other fluids from being poured into his mouth, and he has no difficulty of swallowing. He likewise complains of pain along the spine, and that sometimes he is drawn backward by a sudden spasm; he is likewise afflicted with pain of his left arm, and of that side of the neck with slight spasmodic twitches. Pulse quick, small, but very regular; skin natural; body very costive; some thirst; pain of head and of belly; his jaw and neck have been rubbed with liq. vol.

℞. Opii puri ʒj. Axung q. s

Ut fiat unguentum. Divide in partes, vj. Let his jaw be be rubbed with one of these portions for a quarter of an hour. Let this be done six times in the course of the day.

Habt. Pulv. Jalap gr. xxv.

Let him have broths and beer.

April 29. The pains of his back and the spasmodic contractions have somewhat abated; and the jaw is not so fast locked. The opium was well rubbed in, but produced no inclination to sleep. Body costive.

Rep. Frictio Opiata. Hab. Pil. Opii. ij. bis in die.

May 2. The medicines ordered to be repeated, with the addition of the electuarium purgans of the Dispensary; and, if that should not operate, the administration of a clyster.

May 4. Trismus as before; severe pain of bowels, with extreme costiveness. The clyster had very little effect; the electuary none at all. Good sleep since he took the pills; pulse small and quick; no difficulty in swallowing.

Rep. Frictio Opiata. Habt. Mist. Cathart. of the Dispensary, coch. mag. ij. tertia quaque hora.

May 5. Spasm of jaw somewhat relaxed; still he complains of great rigidity of his limbs and back; took about 3 oz. of mist. cathart.; had a copious stool this morning, and another at noon; slept well last night. He began to take the bark to day. Contin.

Trismus gradually abating; his limbs are not so stiff; pain of neck and back relieved; body open; he can sit up for some hours together; good sleep. Contin. med.

May 8. Jaw cannot yet be completely opened, but he recovers strength and appetite; pains relieved; body open; stiffness of limbs abating; let him have wine and broths.

Rep. M. Cinchon. & Frictio Opiata.

May 9. Trismus as before; recovers strength; complains of pain in his thigh.

Contin. Med. Cinchon. et Frictio Opiata. Frict. femur lin. volat.

May 13. Trismus gone; still a slight stiffness of jaws and of his knees.

Om. Frictio Opiata. Con. lin. vol. et mist. peruv.

May 16. Stiffness of jaws gone; knees still troublesome; continues to recover strength. Rep.

May 19. Decidedly convalescent; let him continue the wine. Rep. mist. peruv.

May 23. Cured.

During the process of convalesence, in addition to the bark, steel was prescribed according to the following formula.

Ferri ppt. gr. v. G. myrrh. gr. x. G. Arab. q. s. Fiat mass. divid. in pil. iij. Capiat æger pil. j. ter in die.

